# Hemoglobin A1c as screening for gestational diabetes mellitus in Nordic Caucasian women

**DOI:** 10.1186/s13098-016-0168-y

**Published:** 2016-07-22

**Authors:** Ingrid Hov Odsæter, Arne Åsberg, Eszter Vanky, Siv Mørkved, Signe Nilssen Stafne, Kjell Åsmund Salvesen, Sven Magnus Carlsen

**Affiliations:** Department of Clinical Chemistry, Clinic of Laboratory Medicine, St. Olavs Hospital, Trondheim University Hospital, Trondheim, Norway; Department of Obstetrics and Gynecology, Women’s Clinic, St. Olavs Hospital, Trondheim University Hospital, Trondheim, Norway; Department of Clinical Services, St. Olavs Hospital, Trondheim University Hospital, Trondheim, Norway; Department of Endocrinology, Clinic of Medicine, St. Olavs Hospital, Trondheim University Hospital, Trondheim, Norway; Department of Cancer Research and Molecular Medicine, Faculty of Medicine, Norwegian University of Science and Technology, Trondheim, Norway; Department of Laboratory Medicine, Children’s and Women’s Health, Faculty of Medicine, Norwegian University of Science and Technology, Trondheim, Norway; Department of Public Health and General Practice, Faculty of Medicine, Norwegian University of Science and Technology, Trondheim, Norway

**Keywords:** Birth weight, Gestational diabetes mellitus, HbA1c, Preeclampsia, Screening

## Abstract

**Background:**

Gestational diabetes mellitus (GDM) increases the risk for preeclampsia and macrosomia. GDM is conventionally diagnosed by an oral glucose tolerance test (OGTT). Hemoglobin A1c (HbA1c) is a marker for the average glucose level the last 2–3 months. We aimed to study if HbA1c alone or in combination with patient characteristics can be used to screen for GDM and reduce the number of OGTTs, and whether it could predict preeclampsia or birth weight.

**Methods:**

855 women from a previous study on the effect of exercise on GDM prevalence were eligible, whereof 677 were included. GDM was diagnosed by WHO 1999 criteria (GDM-WHO) and modified IADPSG criteria (GDM-IADPSG), at pregnancy weeks 18–22 and 32–36. HbA1c analyzed at pregnancy weeks 18–22 and 32–36, variables from patient history and clinical examination were considered for logistic regression models. The diagnostic accuracy was assessed by ROC curve analysis.

**Results:**

Accumulated GDM prevalence was 6.7 % by WHO and 7.2 % by modified IADPSG criteria. Nearly a third could potentially have avoided an OGTT by using HbA1c to *exclude* GDM-IADPSG with a sensitivity of 88 % at week 18–22 and 97 % at week 32–36. Further, 16 % could have avoided an OGTT with a sensitivity of 96 % using HbA1c at week 18–22 to *exclude* GDM-IADPSG throughout pregnancy. HbA1c was not accurate at *diagnosing* GDM-IADPSG, and it was inaccurate at screening for GDM-WHO at any time point. Adding other predictors did not increase the number of potentially avoidable OGTTs significantly. HbA1c was not significantly associated with preeclampsia or birth weight.

**Conclusions:**

HbA1c could potentially reduce the number of OGTTs.

## Background

Women with gestational diabetes mellitus (GDM) have an increased risk of obstetrical complications and adverse pregnancy outcomes such as preeclampsia and macrosomia [1]. According to a review, GDM prevalences of 2–6 % were most often reported in Europe [2]. GDM is usually diagnosed by an oral glucose tolerance test (OGTT) [2]. However, the OGTT is time-consuming for both the women and the health care system as the women need to be fasting and wait for 2 h to complete the test [2], and an OGTT may induce or aggravate nausea and vomiting in pregnant women, i.e. some fail to complete the test.

There is no international consensus on screening for GDM. Some European countries recommend screening all pregnant women with OGTT, whereas others use selective screening based on risk factors or a glucose challenge test [2]. In 2010 new criteria and universal screening for GDM was suggested by the International Association of Diabetes and Pregnancy Study Groups (IADPSG), i.e. the IADPSG criteria [3]. So far only a few studies on screening tests for the IADPSG criteria are published [4].

Over the last years, hemoglobin A1c (HbA1c), a marker representing the average of plasma glucose level in the last 8–12 weeks [5], has been endorsed as a diagnostic marker for diabetes mellitus in non-pregnant subjects [6]. HbA1c has advantages compared to the OGTT as the blood sample can be drawn in a non-fasting state and there is no need for glucose ingestion or timed blood sampling [6]. Further, the sample stability is better for HbA1c than for plasma glucose [6]. Studies examining HbA1c as a screening test for GDM have been published [7–13]. They are difficult to compare, especially due to different diagnostic criteria for GDM, and no obvious diagnostic threshold has been identified [4]. More research is needed on HbA1c as a screening tool for GDM [14]. We aimed to investigate if HbA1c at pregnancy weeks 18–22, or 32–36 or HbA1c in combination with other clinical data could be used to screen for GDM and potentially reduce the number of OGTTs. Further, we wanted to examine if HbA1c could predict preeclampsia and birth weight.

## Methods

### Study population

We used data from a previously reported randomized controlled trial comparing the effect of a 12-week regular exercise program to standard antenatal care on GDM prevalence [15]. Pregnant women booking an appointment for ultrasound scan in gestational week 18 at St. Olavs Hospital (Trondheim University Hospital) from April 2007 to June 2009 and Stavanger University Hospital from October 2007 to January 2009 were invited to participate. More than 97 % of pregnant Norwegian women attend a free of charge ultrasound scan around week 18. During the study period around 12,000 women had routine ultrasound scans at the two study centers and were eligible for the study. Inclusion criteria were age ≥18 years and a singleton viable fetus. Exclusion criteria were high-risk pregnancies or diseases that could interfere with participation. In addition, women who lived more than 30 min drive from the study center were excluded due to practical reasons. A total of 875 women consented to participate, whereof 20 were excluded due to twin pregnancies (n = 2), miscarriages (n = 5) and not meeting inclusion criteria (n = 13). Data was collected at inclusion (week 18–22) and follow-up (week 32–36). All participants gave written informed consent. The study was approved by the Committee for Medical Research Ethics of Health Region IV in Norway (REK 4.2007.81) and is registered at http://www.clinicaltrials.gov as NCT 00476567. The Declaration of Helsinki was followed throughout the study.

Women in the intervention group received a standardized exercise program of 60 min duration including aerobic activity, strength training and balance exercises instructed by a physiotherapist once a week over a 12-week period. They were encouraged to follow a 45 min home exercise program (30 min endurance training and 15 min strength and balance exercises) at least twice a week. Women in the control group received standard antenatal care and the customary information given by their midwife or general practitioner. They were not discouraged from exercising on their own. Women in both groups received written recommendations on diet, pelvic floor muscle exercises, and pregnancy-related lumbo-pelvic pain.

Women diagnosed with GDM by the World Health Organization (WHO) criteria from 1999 [16] during the study period received standard treatment for GDM, i.e. initially diet and life style advice. Insulin treatment was considered if serum glucose (s-glucose) was persistently elevated, i.e. fasting s-glucose >6.0 mmol/L or >8.0 mmol/L 1–1.5 h after a meal. None of the participants needed insulin treatment.

### Clinical data

Age, smoking status and information regarding previous pregnancies and family history of diabetes were collected through questionnaires. Macrosomia was defined as birth weight >4000 g. Weight, height and blood pressure were measured at pregnancy weeks 18–22. After 15 min rest, blood pressure was measured three times with 2 min break between measurements. The average of the two last measurements was used. Data on pregnancy complications and adverse outcomes were obtained from medical records. Preeclampsia was defined as systolic blood pressure over 140 mmHg and/or diastolic blood pressure over 90 mmHg and proteinuria ≥0.3 g/24 h measured more than once 4–6 h apart occurring after gestational week 20.

### Diagnostic criteria

GDM was diagnosed at pregnancy weeks 18–22 and 32–36 by the WHO criteria from 1999 as fasting s-glucose ≥7.0 mmol/L or s-glucose ≥7.8 mmol/L 2 h after ingesting 75 g glucose orally (OGTT) [16]. After the study we also diagnosed GDM according to *modified* IADPSG criteria as fasting s-glucose ≥5.1 mmol/L or s-glucose ≥8.5 mmol/L 2 h after the glucose load [3]. We could only use *modified* IADPSG criteria since we did not have 1-h s-glucose. GDM diagnosed by the WHO criteria will hereafter be named GDM-WHO and by *modified* IADPSG criteria will be named GDM-IADPSG.

### Laboratory analyses

Fasting and 2-h glucose levels were measured in serum by the routine methods used by the hospital laboratory.

Venous blood samples from pregnancy weeks 18–22 and 32–36 were stored at −80 °C. HbA1c is stable at these storage conditions [17, 18]. HbA1c was analyzed over a 3-week period in October 2014 at our hospital laboratory with an immunological method on a Roche Cobas Integra (Roche Diagnostics, Mannheim, Germany) [19]. The method was calibrated against the standard from the International Federation of Clinical Chemistry and Laboratory Medicine [20]. The coefficient of variation for within-laboratory imprecision during the 3-week period was 2.0 % at HbA1c 5.2 % (33 mmol/mol) and 1.4 % at HbA1c 9.6 % (81 mmol/mol) [21].

### Statistical analyses

To evaluate if HbA1c could potentially reduce the number of OGTTs we used a diagnostic threshold with high sensitivity to rule-out GDM and a threshold with high specificity to rule-in GDM. Those between the two thresholds would need an OGTT to clarify whether they had GDM or not. We evaluated if HbA1c at weeks 18–22 could predict GDM at 18–22 weeks of pregnancy, and if it could predict GDM throughout pregnancy, i.e. GDM diagnosed at weeks 18–22 and 32–36 combined. We also evaluated if HbA1c at pregnancy weeks 32–36 could predict GDM at weeks 32–36. To evaluate HbA1c as a predictor for GDM and preeclampsia together with other data, we used logistic regression with backwards elimination to find the best combination of variables in predicting the outcome. We used receiver operating characteristic (ROC) curve analysis to evaluate the diagnostic accuracy of HbA1c alone, the models predicting GDM and to find suitable diagnostic thresholds for not performing an OGTT [22]. We also assessed goodness-of-fit for the models by the Hosmer–Lemeshow test. The leverage of individual points was visually judged by inspecting a plot of $\Delta \hat{\beta }_{i}$ against *p*, where $\Delta \hat{\beta }_{i}$ is the amount that the logistic regression model parameters change when the ith observation is omitted from the model, and *p* is the estimated probability of the outcome.

To find the best combination of variables predicting birth weight, we used linear regression. Model assumptions and fit and identification of observations with potentially high influence on the model were evaluated by inspection of residual plots, R^2^ and the DFBETA statistic which quantifies how much the regression coefficients change when the ith observation is omitted from the model.

For all regression analyses we used the Royston and Altman algorithm to find the simplest (if any) non-linear transformation of the continuous variables [23]. The selection of variables was based on variables that should be easily available to the clinician at the time point of HbA1c testing and known to be associated with or suspected to influence on the dependent variable. Also, we included the intervention as a possible predictor variable in all models for GDM at week 32–36 and throughout pregnancy, preeclampsia and birth weight in order to adjust for possible confounding. The significance level for keeping a variable in the model was *p* value ≤0.10.

We used bootstrap analysis to assess the stability of the multivariable models where the bootstrap inclusion fraction is an indicator for the importance of each independent variable [24].

The statistical analyses were performed using Stata version 13.1 for Windows (Stata Corp., Texas, USA).

## Results

**Fig. 1:**
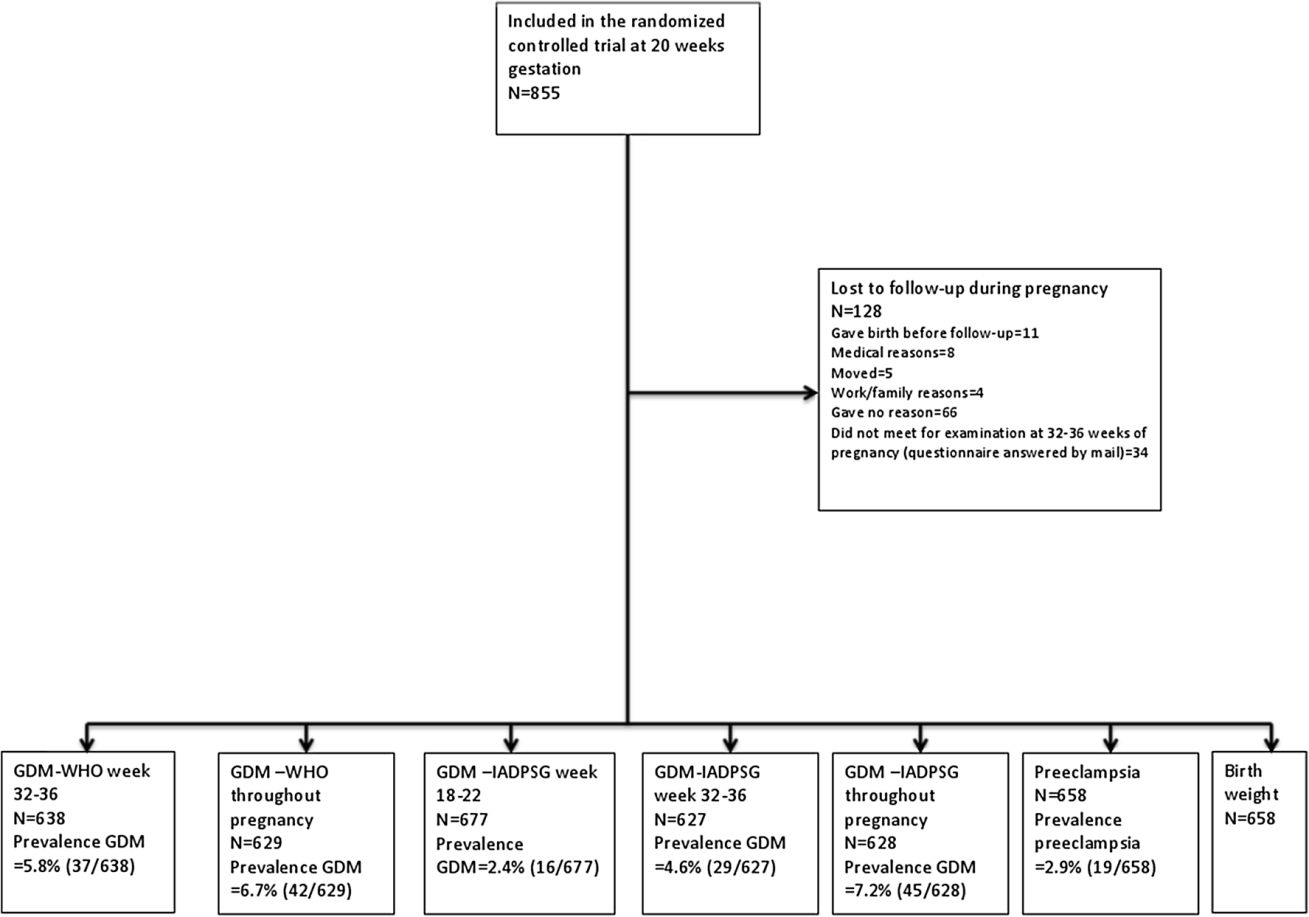
Flow diagram of the study participants. The information in the *lowest row* shows how many of the study participants that had a complete data set for all potential predictors considered in the model, i.e. the number of participants included in the analyses for HbA1c alone and for HbA1c together with other data at that time point

**Fig. 2:**
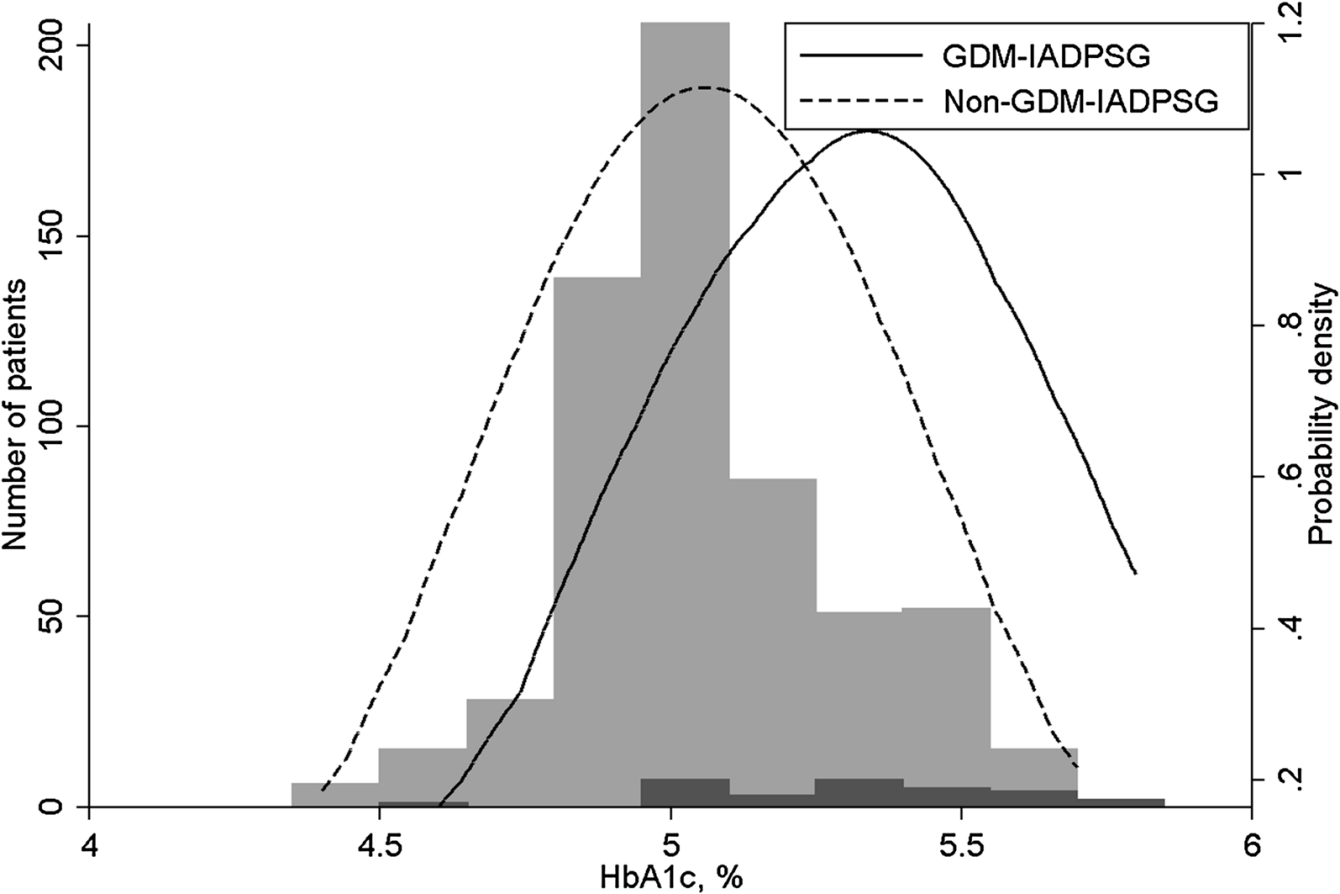
The distribution of HbA1c at pregnancy weeks 32–36 in those diagnosed with gestational diabetes mellitus (*black columns*) and not (*grey columns*) at gestational week 32–36 by the modified IADPSG criteria. In addition to the histograms, the figure shows a kernel density plot of HbA1c in each group, where the distributions are smoothed and scaled to the same level of probability density

In all, 855 women were included in the study. One hundred twenty-eight were lost to follow-up, 25 did not complete the OGTT at pregnancy weeks 18–22, and 25 did not complete an OGTT at pregnancy weeks 32–36. Due to missing data 627–677 women were included in the analyses (Fig. 1 and Table 1). Women with missing data were more often smokers (2.4 vs 0.5 %, p = 0.02) and had slightly higher systolic blood pressure (median 110 vs 108 mmHg, p = 0.03) and body mass index (BMI) (median 24.7 vs 24.2 kg/m^2^, p = 0.01). Table 1 lists characteristics of the study population. The distribution of HbA1c at gestational week 32–36 among those diagnosed with and without GDM by the IADPSG criteria at gestational week 32–36 is shown in Fig. 2.

**Table 1:** Characteristics of the study population

	N	Median (min–max) or n (%)
Age (years)	839	30 (19–46)
BMI (kg/m^2^)	854	24.3 (18.4–39.9)
Education at university college or university level	855	753 (88.1)
Exercised at moderate to high intensity at least three times per week prior to pregnancy	855	269 (31.5)
Nulliparity	853	485 (56.9)
Family history of diabetes	716	64 (8.9)
GDM in previous pregnancy	855	4 (0.4)
Previous macrosomic baby	855	70 (8.2)
Intervention group	855	429 (50)
Smoking in week 18–22	855	9 (1.1)
Systolic blood pressure week 18–22 (mmHg)	855	109 (82–147)
HbA1c week 18–22 [%, (mmol/mol)]	845	4.8 (4.2–5.7) [29 (22–39)]
HbA1c week 32–36 [%, (mmol/mol)]	722	5.1 (4.4–5.8) [32 (25–40)]
Fasting s-glucose week 18–22 (mmol/L)	849	4.3 (3.4–5.6)
2-h s-glucose week 18–22 (mmol/L)	836	4.8 (2.1–10.1)
Fasting s-glucose week 32–36 (mmol/L)	711	4.3 (3.2–6.4)
2-h s-glucose week 32–36 (mmol/L)	702	5.6 (2.3–9.9)
Birth weight (g)	853	3540 (825–4930)

### HbA1c as a screening test for GDM-WHO

It was impossible to assess HbA1c as a screening test for GDM-WHO around 20 weeks of pregnancy, since only five (0.7 %) women tested positive for GDM-WHO at pregnancy weeks 18–22. Between the 32nd and the 36th weeks of gestation, 37 (5.8 %) women were diagnosed with GDM-WHO and throughout pregnancy (i.e. diagnosed at pregnancy weeks 18–22 or 32–36) 42 (6.7 %) women were diagnosed with GDM-WHO.

The area under the ROC curve for HbA1c in diagnosing GDM-WHO at pregnancy weeks 32–36 was 0.74 (95 % CI 0.64–0.83). HbA1c at 32–36 weeks of pregnancy, age and BMI at inclusion, family history of diabetes mellitus (yes/no), GDM in previous pregnancy (yes/no), previously giving birth to a macrosomic baby (yes/no) and intervention (i.e. being in the exercise group or control group) were potential predictors for the model for GDM-WHO at pregnancy weeks 32–36. Only HbA1c and age were included in the model (Table 2), with an area under the ROC curve of 0.76 (95 % CI 0.66–0.85). Sensitivity and specificity for diagnosing GDM at various levels of HbA1c and the predicted probability of GDM from the model are presented in Table 3.

**Table 2:** Odds ratio, 95 % confidence interval and p-value for predictors for GDM-WHO at week 32–36, GDM-WHO diagnosed at week 18–22 or 32–36, GDM-IADPSG at week 18–22, GDM-IADPSG at week 32–36 and GDM-IADPSG diagnosed at week 18–22 or 32–36

Predictor	GDM-WHO week 32–36			GDM-WHO throughout pregnancy			GDM-IADPSG week 18–22^a^			GDM-IADPSG week 32–36^a^			GDM-IADPSG throughout pregnancy^a^		
	OR	95 % CI	p-value	OR	95 % CI	p-value	OR	95 % CI	p-value	OR	95 % CI	p-value	OR	95 % CI	p-value
HbA1c week 18–22				10.1	2.1–49	0.004	11.4	1.10–119	0.04				21	4.6–99	<0.0005
HbA1c week 32–36	49	11–215	<0.0005							56	11–291	<0.0005			
Age	1.08	1.00–1.17	0.05	1.08	1.00–1.16	0.06									
Family history of diabetes				2.31	1.00–5.3	0.05									
BMI							1.13	0.98–1.30	0.09	1.12	0.99–1.26	0.06	1.15	1.05–1.26	0.002

The ORs are for one percentage point increase in HbA1c, one year increase in age and a unit (kg/m^2^) increase in BMI

^a^Modified IADPSG criteria were used, i.e. 1-h s-glucose was missing

**Table 3:** Sensitivities and specificities around 95, 97.5 and 100 % for diagnosing GDM with corresponding cut-offs for HbA1c and probabilities for GDM estimated by logistic regression models

Outcome	HbA1c alone^a^					Model				
	Cut-off, HbA1c ≥ (%)	Sensitivity (%)	Specificity (%)	Percentage of women avoiding OGTT	Percentage of women avoiding OGTT misclassified	Cut-off, probability of GDM ≥	Sensitivity (%)	Specificity (%)	Percentage of women avoiding OGTT	Percentage of women avoiding OGTT misclassified
GDM-WHO week 32–36	4.7	100	3.7	3.4	0	0.009	100	8.5	8.0	0
	4.8	97.3	8.0	7.7	2.0	0.011	97.3	11.2	10.7	1.5
	4.9	91.9	17.1	16.6	2.8	0.013	94.6	14.3	13.8	2.3
	5.5	29.7	95.0	6.4	73.2	0.16	29.7	95.0	6.4	73.2
	5.6	21.6	97.7	3.4	63.3	0.22	21.6	97.7	3.4	63.3
	5.9	0	100	0	0	0.50	0	100	0	0
GDM-WHO throughout pregnancy	4.4	100	0.5	0.5	0	0.018	100	2.9	2.7	0
	4.5	97.6	2.4	2.4	6.7	0.026	97.6	11.2	10.6	1.5
	4.6	95.2	7.7	7.5	4.3	0.027	95.2	11.8	11.3	2.8
	5.2	14.3	95.2	5.4	82.4	0.15	19.1	95.1	5.9	78.4
	5.3	9.5	97.8	2.7	76.4	0.18	16.7	97.4	3.5	68.6
	5.8	0	100	0	0	0.37	2.4	100	0.2	0
GDM-IADPSG week 18–22^b^	4.7	100	16.6	16.2	0	0.010	100	16.0	15.7	0
	4.8	87.5	30.3	29.8	1.0	0.0153	93.8	36.0	35.3	0.4
	4.9	68.8	51.3	50.8	1.5	0.0154	87.5	36.5	35.9	0.8
	5.2	12.5	94.7	5.5	94.6	0.056	18.8	95.0	5.3	91.7
	5.3	6.3	97.4	2.7	94.5	0.067	0	97.4	2.5	100
	5.8	0	100	0	0	0.18	0	100	0	0
GDM-IADPSG week 32–36^b^	4.6	100	1.0	1.0	0	0.003	100	2.0	1.9	0
	5.0	96.6	31.4	30.1	0.5	0.013	96.6	23.9	23.0	0.7
	5.1	82.8	48.2	46.7	1.7	0.018	93.1	36.0	34.6	0.9
	5.5	31.0	94.7	6.5	77.9	0.13	37.9	95.0	6.5	73.2
	5.6	20.7	97.5	3.3	71.4	0.18	24.1	97.5	3.5	68.2
	5.8	6.9	100	0.3	0	0.57	3.5	100	0.2	0
GDM-IADPSG throughout pregnancy^b^	4.4	100	0.5	0.5	0	0.014	100	3.3	3.0	0
	4.6	97.8	2.4	2.4	6.6	0.027	97.8	16.6	15.6	1.0
	4.7	95.6	16.5	15.6	2.0	0.037	95.6	30.0	28.2	1.1
	5.2	13.3	95.2	5.4	82.3	0.18	26.7	95.0	3.2	75.2
	5.3	8.9	97.8	2.7	76.2	0.21	11.1	97.4	6.5	70.7
	5.8	0	100	0	0	0.52	0	100	0	0

See Table 2 for variables included in the various models. In addition, the percentage of women avoiding an OGTT by using cut-offs with high specificity to rule-in and high sensitivity to rule-out GDM and the percentage of those avoiding OGTT who are misclassified are shown

^a^HbA1c at gestational week 18–22 was used for GDM-WHO throughout pregnancy, GDM-IADPSG at week 18–22 and GDM-IADPSG throughout pregnancy, while HbA1c at gestational week 32–36 was used for GDM-WHO at week 32–36 and GDM-IADPSG at week 32–36

^b^Modified IADPSG criteria were used, i.e. 1-h s-glucose was missing

For HbA1c at week 18–22 in diagnosing GDM-WHO throughout pregnancy, the area under the ROC curve was 0.64 (95 % CI 0.55–0.72). HbA1c at week 18–22, age, BMI, family history of diabetes mellitus, GDM in previous pregnancy, previously giving birth to a macrosomic baby and intervention were potential predictors for the model for GDM-WHO throughout pregnancy. Predictors included in the model were HbA1c, age and a family history of diabetes (Table 2). The area under the ROC curve for the model was 0.67 (95 % CI 0.58–0.76). Table 3 presents sensitivity and specificity for diagnosing GDM at potential cut-offs for HbA1c and the predicted probability of GDM from the model.

None of those with GDM-WHO were diagnosed because of an elevated fasting s-glucose. With only 2-h s-glucose ≥7.8 mmol/L as the outcome, fasting s-glucose had about the same diagnostic accuracy and ability to potentially reduce the number of OGTTs as HbA1c. Including fasting s-glucose in the models predicting a 2-h s-glucose ≥7.8 mmol/L did not improve the screening ability significantly.

### HbA1c as a screening test for GDM-IADPSG

At pregnancy weeks 18–22, 32–36 and throughout pregnancy (i.e. at week 18–22 or 32–36) 16 (2.4 %), 29 (4.6 %) and 45 (7.2 %) women were diagnosed with GDM-IADPSG, respectively.

The area under the ROC curve for HbA1c in diagnosing GDM-IADPSG at pregnancy weeks 18–22 was 0.67 (95 % CI 0.54–0.80). HbA1c at 18–22 weeks of pregnancy, age, BMI, family history of diabetes mellitus, GDM in previous pregnancy and previously giving birth to a macrosomic baby were potential predictors for the model for GDM-IADPSG at pregnancy weeks 18–22. HbA1c and BMI were the only predictors included in the model (Table 2), with an area under the ROC curve of 0.70 (95 % CI 0.57–0.84). Potential cut-offs for HbA1c and the predicted probability of GDM with corresponding sensitivity and specificity for diagnosing GDM are presented in Table 3.

HbA1c had an area under the ROC curve of 0.76 (95 % CI 0.67–0.85) in diagnosing GDM-IADPSG at 32–36 weeks of pregnancy. HbA1c at weeks 32–36, age, BMI, family history of diabetes mellitus, GDM in previous pregnancy, previously giving birth to a macrosomic baby and intervention were potential predictors for the model for GDM-IADPSG at pregnancy weeks 32–36. HbA1c, BMI and intervention were the predictors included in the model. We assessed possible interaction between HbA1c and intervention, but the interaction term was not statistically significant (p = 0.71), and we excluded the intervention variable from the model. The area under the ROC curve for the model with HbA1c and BMI as predictors (Table 2) was 0.77 (95 % CI 0.68–0.87). Table 3 shows sensitivity and specificity for diagnosing GDM at potential cut-offs for HbA1c and the predicted probability of GDM estimated by the model.

The area under the ROC curve for HbA1c at pregnancy weeks 18–22 in predicting GDM-IADPSG throughout pregnancy was 0.69 (0.60–0.77). HbA1c at 18–22 weeks of pregnancy, age, BMI, family history of diabetes mellitus, GDM in previous pregnancy, previously giving birth to a macrosomic baby and intervention were potential predictors for the model for GDM-IADPSG throughout pregnancy. HbA1c and BMI were included in the model (Table 2) with an area under the ROC curve of 0.72 (0.64–0.80). Sensitivity and specificity for diagnosing GDM at various levels of HbA1c and the predicted probability of GDM from the model are shown in Table 3.

Only four women had history of GDM in a previous pregnancy and none of them had GDM-IADPSG, so this variable was omitted from the analyses of GDM-IADPSG. We performed the analyses for the multivariable models for GDM-IADPSG without the previous GDM variable, and a sensitivity analyses without those with previous GDM. The results did not change after exclusion of those with previous GDM.

### HbA1c as predictor for preeclampsia

The prevalence of preeclampsia was 2.9 %. Predictor variables considered for the preeclampsia model were HbA1c at 18–22 weeks of pregnancy, age, BMI, smoking (yes/no) and systolic blood pressure at inclusion, intervention, nulliparity and GDM-WHO. We also performed the analyses with GDM-IADPSG as an independent variable instead of GDM-WHO, and with HbA1c at weeks 32–36 instead of at weeks 18–22. Variables included in the model for preeclampsia were GDM-WHO (OR 2.97, 95 % CI 0.82–10.7, p = 0.10) and nulliparity (OR 2.80, 95 % CI 0.92–8.5, p = 0.07). The area under the ROC curve for this model was 0.67 (95 % CI 0.58–0.76). Neither HbA1c at pregnancy weeks 18–22 or 32–36 nor GDM-IADPSG were statistically significantly associated with preeclampsia (p > 0.10).

None of the nine women who smoked had preeclampsia, and this variable was omitted from the analyses of the model for preeclampsia. We therefore performed the analyses without the smoking variable, and a sensitivity analyses without those smoking. The results did not change after exclusion of those who smoked.

### HbA1c as predictor for birth weight

The following predictor variables were considered for the model predicting birth weight; HbA1c at pregnancy weeks 18–22, age, BMI and smoking at inclusion, intervention, nulliparity, previously giving birth to a macrosomic baby and GDM-WHO. We also performed the analyses with GDM-IADPSG as an independent variable instead of GDM-WHO, and with HbA1c at pregnancy weeks 32–36 instead of at weeks 18–22. Variables included in the model for birth weight were HbA1c at pregnancy weeks 32–36 (β 137, 95 % CI −10 to 283, p = 0.07), BMI (β 35, 95 % CI 23–47, p < 0.0005), nulliparity (β −102, 95 % CI −178 to −28, p = 0.008) and previously giving birth to a macrosomic baby (β 301, 95 % CI 161–440, p < 0.0005). Neither HbA1c at pregnancy weeks 18–22, GDM-WHO nor GDM-IADPSG were statistically significantly associated with birth weight (p > 0.10).

We did not find any non-linear associations between the continuous variables and the outcome for any of the models. We assessed the stability of the multivariable models by bootstrap analysis [24]. The results were in consistence with our final models as HbA1c was selected in 74–100 % of the replicates for all GDM models, except for the model predicting GDM-IADPSG at pregnancy weeks 18–22 where it was chosen in 31 % of the replicates. For the other predictors of GDM, only BMI in the model for GDM-IADPSG throughout pregnancy was chosen in more than 60 % of the replicates (79 %).

## Discussion

Around 30 % of pregnant women could potentially have avoided an OGTT by using HbA1c ≤4.8 % (29 mmol/mol) to *exclude* GDM-IADPSG with a sensitivity of 88 % at pregnancy weeks 18–22 and by using HbA1c ≤5.0 % (31 mmol/mol) with a sensitivity of 97 % at pregnancy weeks 32–36 (Table 3). Further, 16 % could potentially have avoided an OGTT with a sensitivity of 96 % by using HbA1c ≤4.7 % (28 mmol/mol) at pregnancy weeks 18–22 to *exclude* GDM-IADPSG throughout pregnancy (Table 3). Adding other variables to predict GDM did not significantly increase the number of potentially avoidable OGTTs. HbA1c was not accurate at *diagnosing* GDM-IADPSG since most of those diagnosed would have been false positives (Table 3). HbA1c was inaccurate at screening for GDM-WHO at any time point. Neither HbA1c nor GDM was accurate in predicting preeclampsia or birth weight.

Agarwal et al. studied HbA1c as a screening test for GDM by WHO 1999 criteria in gestational weeks 24–28 [8]. They found an area under the ROC curve of 0.54 (95 % CI 0.48–0.61), significantly lower than our result at pregnancy weeks 32–36, but similar to our result for HbA1c from pregnancy weeks 18–22 in diagnosing GDM-WHO throughout pregnancy. They also found that when high levels of HbA1c were used to diagnose GDM, most positive test results would be false positives. At thresholds they considered had acceptable sensitivity, they could only exclude GDM in a few women. They also concluded that HbA1c is unsuitable for screening for GDM-WHO.

Rajput et al. and Sevket et al. found an area under the ROC curve for HbA1c in diagnosing GDM by IADPSG criteria in pregnancy weeks 24–28 of 0.683 (95 % CI 0.601–0.765) when screening 607 Indian women and 0.697 (95 % CI 0.645–0.745) among 339 women in Turkey, respectively [10, 12], both were comparable to our results. Although the overall diagnostic accuracy for HbA1c in diagnosing GDM by IADPSG criteria was similar for the present and previous studies, the diagnostic thresholds for HbA1c and the sensitivity and specificity at the same levels of HbA1c differ between studies [10, 12]. These differences probably reflect diversities between populations, testing at different gestational lengths and the analytical methods used. However, there are also similarities in the studies: HbA1c is not accurate at diagnosing GDM; around 45 % of those ruled-into GDM would have been false positives in the study of Rajput et al. [10], 67 % in the study of Sevket et al. [12] and more than 60 % in our study (Table 3). Using HbA1c to exclude GDM, 35 % could have potentially avoided OGTT in the study by Rajput et al. with a false negative rate of 17 % [10], and 21 % in the study by Sevket and colleagues with a false negative rate of 4 % [12]. In our study around 30 % could have avoided an OGTT with a false negative rate of 3 % (i.e. sensitivity 97 %) at pregnancy weeks 32–36 (Table 3).

The strengths of our study are the inclusion of other clinical variables in the multivariable prediction models and the possibility to evaluate prediction of GDM by both WHO and modified IADPSG diagnostic criteria.

Our study has limitations. The number of women with GDM was low, and this increases the risk of a type II error. The study participants were healthy, highly-educated, Caucasian women with low-risk pregnancies (Table 1). Our results may not be relevant in populations with higher BMI, less physical activity and education or other ethnicities. Furthermore, only 73–79 % of those originally included in the randomized controlled trial were included in the present analyses. Women lost to follow-up could have led to a further selection of low-risk pregnancies. The differences in smoking rate (2.4 vs 0.5 %, p = 0.02), systolic blood pressure (median 110 vs 108 mmHg, p = 0.03) and BMI (median 24.7 vs 24.2 kg/m^2^, p = 0.01) for those with missing data versus those with complete data could suggest that, however, the absolute differences were small and the clinical significance questionable. It is most common to test for GDM around gestational week 24–28, so our timing of testing is a weakness of the study.

Like some previous studies [25, 26] we used *modified* IADPSG criteria, since 1-h s-glucose was not available in our study. Thus, we cannot exclude misclassification of some women as normal glucose tolerant instead of GDM. Studies indicate that 14–21 % of GDM cases according to IADPSG criteria are diagnosed by the 1-h value alone [27–29]. Accordingly, up to one-fifth of GDM-IADPSG cases may be misclassified as non-GDM-IADPSG. Most probably these women had HbA1c in the upper range. In sensitivity analyses we found that even if 20 % were misclassified and all of them had high HbA1c values, reclassifying them as GDM would not change the figures in Table 3 significantly.

OGTT may be questioned as gold standard. In some studies, 22–24 % of pregnant women were reclassified when the OGTT was measured 1–2 weeks apart [30, 31]. Furthermore, the WHO 1999 criteria are based on risk for diabetes-specific microvascular complications in non-pregnant populations, and the IADPSG criteria are based on an increased risk for birth weight, cord C-peptide and percentage body fat above the 90th percentile [32]. Thus, GDM is more a risk factor than a disease per se. GDM carries a rather small increased risk for pregnancy complications and adverse pregnancy outcomes, and most women diagnosed do not develop complications [33]. In one study, the area under the ROC curve for IADPSG criteria in predicting a composite adverse pregnancy outcome including preeclampsia, large-for-gestational age newborn and perinatal death, was 0.582 (95 % CI 0.559–0.604) [34].

Although HbA1c is not very good at diagnosing GDM, it may have a potential to predict adverse pregnancy outcomes. We found that HbA1c was unable to predict preeclampsia. However, we had few women with preeclampsia (n = 19, 2.9 %) and low power to detect a possible association. Others have found an association between HbA1c and preeclampsia [35, 36].

In the model predicting birth weight, HbA1c at pregnancy weeks 32–36 was included (β 137, 95 % CI −10 to 283, p = 0.07). However, the association was only borderline statistically significant, and in an evaluation of the stability of the model [24], it was only selected in 23 % of the replicates. The HAPO study found that associations with birth weight were significantly stronger for glucose than for HbA1c [35]. Hou et al. found no significant difference in HbA1c at pregnancy weeks 28–37 in non-diabetic women having newborns appropriate-for-gestational age compared to large-for-gestational age [37]. In contrast, Karcaaltincaba and co-workers found a positive and independent association between second trimester HbA1c and birth weight and none between fasting plasma glucose and birth weight in non-diabetic pregnancies [38]. Hughes et al. found that a high HbA1c before pregnancy week 20 was associated with an increased risk of large-for-gestational age newborn, but not macrosomia [36]. However, a high HbA1c was associated with an increased risk of major congenital anomaly, preeclampsia, shoulder dystocia, and perinatal death [36].

Those diagnosed with GDM-WHO in the present study received standard treatment for GDM, i.e. diet and life style advice, and could thereby have prevented adverse outcomes. This may be an explanation for no or weak associations between the predictor variables and the adverse pregnancy outcomes.

## Conclusions

HbA1c may have a potential for screening for GDM since it is possible to exclude GDM in a significant proportion of women. Whether HbA1c alone or combined with other data can be useful in predicting adverse pregnancy outcomes among normal healthy women is unclear, and more research is needed.

## Abbreviations

BMI: body mass index
CI: confidence interval
GDM: gestational diabetes mellitus
HAPO: hyperglycemia and adverse pregnancy outcome
HbA1c: glycated hemoglobin A1c
IADPSG: The International Association of the Diabetes and Pregnancy Study Groups
NGT: normal glucose tolerance
OGTT: oral glucose tolerance test
OR: odds ratio
ROC: receiver operating characteristic
s-glucose: serum glucose
WHO: World Health Organization
